# Assessing the Degree of Acute Esophageal Injury Secondary to Corrosive Intake: Insights From a Public Sector Hospitals of a Developing Country

**DOI:** 10.7759/cureus.10858

**Published:** 2020-10-08

**Authors:** Qurat Ul Ain, Manahil Jamil, Hafiz Abu Safian, Tayyab S Akhter, Salma Batool, Moniba Arshad, Ali Murad Jamal, Ather Iqbal, Laraib Arsh, Bilawal Abbas

**Affiliations:** 1 Psychology, Rawalpindi Medical University, Rawalpindi, PAK; 2 Surgery, Rawalpindi Medical University, Rawalpindi, PAK; 3 Gastroenterology, Holy Family Hospital, Islamabad, PAK; 4 Internal Medicine, Rawalpindi Medical University, Rawalpindi, PAK

**Keywords:** caustics, alkalies, esophageal mucosa

## Abstract

Background

Caustic ingestion continues to be a significant problem worldwide especially in developing countries and particularly in the age group of under six years. Ingestion of caustic substances is a medical emergency in both the adult and pediatric population and is associated with high morbidity and mortality. The ingestion of caustic substances induces an extensive spectrum of injuries to the aerodigestive tract, which includes extensive necrosis and perforation of the esophagus and stomach.

Objectives

The main aims were to determine upper and lower esophageal injuries associated with corrosive intake and to compare esophageal injury with age and gender.

Rationale

Once we’ll find the extent and severity of esophageal injury associated with corrosive intake within 24 hours, we’ll be able to manage the case timely and to limit further complications and disabilities.

Materials and Methods

This descriptive cross-sectional study was conducted on 150 patients who presented with corrosive ingestion and underwent urgent endoscopic evaluation. Data were collected using self-designed pro forma. Endoscopic findings were classified according to the Zargar classification. A descriptive analysis of study variables was performed using SPSS v.21.0 (IBM Corp., Armonk, NY, USA). The chi-square test was used, and a p-value of less than 0.05 was considered statistically significant.

Results

Out of 150 patients under study, 103 (68.7%) were females and 47 (31.3%) were males. The most prevalent age group presenting with corrosive intake was found to be between 21 and 34 years of age (43.3%) in both genders. The most common part of the esophagus prone to corrosive insult is the upper esophagus (99.3%), whereas, regarding severity, the lower esophagus has more severe injuries (predominant being stage 2B, i.e., 32%). There are no statistically significant differences in esophageal injuries in different age groups (upper esophageal injury: 0.319; lower esophageal injury: 0.696) and genders (upper esophageal injury: 0.769; lower esophageal injury: 0.752).

Conclusions

Most of the patients under study belong to the female gender and teen and younger age group. The predominant upper esophageal injury as a result of corrosive intake is stage 0 injury, and the least common is found to be stage 1 injury. The predominant lower esophageal injury as a result of corrosive intake is stage 2B injury, whereas the least common is found to be stage 4 injury.

## Introduction

Corrosive ingestion includes the ingestion of substances with extremes of pH and is a grave public health problem across the globe. It can be seen in both developed and developing countries, but it is more common in developing countries [1,2]. Sulphuric acid, hydrochloric acid, oxalic acid, sodium hydroxide, potassium hydroxide, and bleaches are some of the most commonly used corrosive materials and are found in drain cleaners, various cleaning agents, dishwasher detergents, hair relaxers, and disk batteries [3]. These substances are readily available and have a loose regulatory control on their production [1]. Ingestion of such substances may cause necrosis of the aerodigestive tract, perforation of the esophagus and stomach, stricture formation, septicemia, and even death [4-7]. Early evaluation of these challenging clinical situations is required as they result in life-long complications [5].

Corrosive intake invariably results in esophageal injury, which can be characterized as acute and chronic depending upon the onset [8,9]. The acute stage is serious and begins minutes after caustic ingestion, and it causes mucosal injury. Congestion and necrosis occur as a result [1]. The pH of a substance, its concentration, its type, the ingested amount, and the duration of ingestion determine the level of severity of esophageal injury [8,9]. Ingestion of caustic substances may result in stricture formation in most cases, and this further results in the inability to swallow food [5]. Rapid assessment of esophageal injuries is essential to prevent progression to esophagogastric perforation or stricture formation [1]. Such early evaluation is important because it can potentially ensure a suitable clinical intervention and limit the extent of the injury.

In developing countries, the incidence of corrosive ingestion is significantly higher and, in most cases, remains unreported [2]. Their actual prevalence cannot be assumed from random observations and personal judgments of healthcare professionals [10]. Various chemical products stored in homes are the source of accidental or intentional exposures that can be observed in people of different ages [11,12]. Different factors including economic, social, and educational factors can affect the prevalence of these accidents.

The focus of our study is to assess acute esophageal injuries presenting within 24 hours of corrosive intake based on the Zargar classification. Corrosive injury has high morbidity and mortality rates since initial evaluation triage and medical management are highly challenging [13]. Due to the concentration of such incidents in developing countries and their low reporting rate, there is a lack of extensive research on the subject. The authors could not find any related discussion on an immediate evaluation method of esophageal injuries after corrosive ingestion. A standard management plan for such patients needs to be developed to prevent irreversible complications. By reporting the patterns of acute upper and lower esophageal injuries and their relation to various patient parameters, the results of this study can revise patient management, ensure timely intervention, and lead to better prognosis and fewer complications in such cases. Immediate recognition and management of the injury leads to a favorable outcome for the patient.

## Materials and methods

Study design

This descriptive cross-sectional study was conducted from January 2018 to January 2019. A total of 150 patients who presented with corrosive ingestion and underwent urgent endoscopic evaluation, that is, within 24 hours of corrosive intake in teaching hospitals (i.e. Holy Family Hospital, Benazir Bhutto Hospital, and District Headquarters Hospital) of Rawalpindi Medical University were included in the study population. Data were collected using self-designed pro forma containing demographic details including age, gender, and history of corrosive ingestion. Endoscopic findings of esophageal injuries were classified according to the Zargar classification depending on the depth and extent of burns. Patients with the esophageal injury not due to acid or alkali ingestion were excluded from the study.

Data analysis

A descriptive analysis of study variables was performed using SPSS Version 21.0 (IBM Corp., Armonk, NY, USA). Cronbach's alpha of the questionnaire was calculated for 75 responses to assess its reliability in our population. It was 0.683, indicating good interscale reliability. Chi-square test was used, and p-value of less than 0.05 was considered statistically significant.

## Results

The study population consisted of 150 patients. The study focused on the patients who presented in emergency with corrosive intake and underwent an endoscopy within 24 hours of corrosive ingestion. The demographic details of the patients are presented in Table 1.

**Table 1:** Demographic details of the patients.

Age	Gender		Total
	Male	Female	
Up to 20 years	15	31	46
21–34 years	20	45	65
35–54 years	09	21	30
55 years or above	03	06	09
Total	47	103	150

The most common part of the esophagus prone to corrosive insult was the upper esophagus (99.3% of the total cases presented with corrosive intake had upper esophageal injury), whereas regarding severity, the lower esophagus has more severe injuries (predominant being stage 2B, i.e., 32%).

The most prevalent upper esophageal injury due to corrosive intake observed with endoscopy performed within 24 hours of intake was found to be stage 0 (48%), the second most common was found to be stage 2A (24%), and the least common was found to be stage 1 (2%).

The most prevalent lower esophageal injury due to corrosive intake observed with endoscopy performed within 24 hours of intake was found to be stage 2B (32%), the second most common was found to be stage 0 (26%) and the least common was found to be stage 4 (0.7%).

Then we correlated age and gender with upper and lower esophageal injuries, respectively. There was statistically no significant relation of esophageal injury with different age groups (age and upper esophageal injury p-value: 0.297; age and lower esophageal injury p-value: 0.568) and genders (gender and upper esophageal injury p-value: 0.768; gender and lower esophageal injury p-value: 0.807). These correlations are summarized in Tables 2, 3. 

**Table 2:** Cross-tabulation of gender with different stages of Zargar classification.

		Zargar classification							Esophageal injury
		Stage 0	Stage 1	Stage 2A	Stage 2B	Stage 3A	Stage 3B	Stage 4	
Gender	Male	25	00	12	05	01	04	00	Upper
		12	00	10	16	05	04	00	Lower
	Female	47	03	25	11	04	13	00	Upper
		29	03	20	36	06	08	01	Lower
Total		113	06	67	68	16	29	01	

**Table 3:** Cross-tabulation of age groups with different stages of Zargar classification.

Age	Zargar classification							Esophageal injury
	Stage 0	Stage 0	Stage 2A	Stage 2B	Stage 3A	Stage 3B	Stage 4	
Up to 20 years	24	01	12	04	00	05	00	Upper
	14	01	10	17	02	02	00	Lower
21-34 years	30	00	19	08	02	06	00	Upper
	17	00	12	23	06	06	01	Lower
35-54 years	14	02	05	03	03	03	00	Upper
	08	02	08	07	03	02	00	Lower
55 years or above	04	00	01	01	00	03	00	Upper
	02	00	00	05	00	02	00	Lower

In all stages, it is obvious that females were more prone to injury. However, only 2% cases presented with stage 1 injury in upper esophageal injury, all of which were females. Similarly, in lower esophageal injury, females were predominant in all stages. All the cases of stage 1 (2%) and stage 4 (0.7%) were females.

Stage 0 was predominant in all age groups, whereas stage 1 was least common with a total of three cases only. Stage 2B was predominant in almost all age groups except for the age group of 35-54 years. Only one case presented with stage 4 injury and that presented in the age group of 21-34 years.

## Discussion

Acute esophageal injuries due to corrosive intake are more prevalent in the upper esophagus, whereas they are more severe in the lower esophagus in most aspects. However, cases of normal upper and lower esophageal mucosa have also been reported with 48.3% of patients having normal upper esophageal mucosa and 28.1% having normal lower esophageal mucosa, which shows that our figures are higher compared to regional countries. In a study conducted in Chandigarh, India, esophageal injuries are found in a majority of the patients (87.8%), gastric injury in 85.4%, and duodenal injury in 34.1% [14]. In our study, the female population with corrosive intake is about twice as that of the male population. In an intercontinental study, statistical analysis of the data indicated higher consumption in Europe and Oceania in boys with a higher average age of years [15]. A study conducted in Tangdu Hospital showed that more men (n = 61) than women (n = 18) ingested caustic substances, with a sex ratio of 3.4:1 during the 30 years [5].

Clinical features depend on the type of substance, amount, physical form, and time of presentation (early or delayed) [16]. In our study, we separately analyzed the severity of the upper and lower esophageal injury, and results are quite different in both the portions. In our study, the predominant stage of upper esophageal injury as a result of corrosive intake is stage 0 injury (normal mucosa) and the least common is stage 1 injury (superficial mucosal edema and erythema). For the lower esophagus, the predominant stage of lower esophageal injury as a result of corrosive intake is stage 2B injury (deep discrete or circumferential ulcer), whereas the least common is stage 4 injury (perforation). The absence of oropharyngeal local changes does not exclude severe esophageal injuries [17]. One extensive study reported on 37% of esophageal injuries of the second- and third-degree in patients who had no apparent oropharyngeal injuries [18]. On the other hand, other studies showed that 70% of patients with severe oropharyngeal injuries did not have significant esophageal post-corrosive burns. Therefore, oropharyngeal injuries are not a reliable indicator of the eventual damages of the esophagus [19].

Another study indicates that patients with oropharyngeal burns do not have significant damage to the esophagus in up to 70%, and, hence, their presence is not a reliable index of esophageal damage [20]. A study comprising 86 patients showed that 25-30 days after corrosive ingestion, 27.8% of the patients developed stenosis presented on the first endoscopic and X-ray control. Of the 86 patients, 25.8% were intoxicated with acids and 66.6% with alkalis. The most common location of the stenosis was the distal part of the esophagus and gastric antrum and pylorus, with 6.4% in both cases. In 16.7% of the patients who ingested alkalis, stenosis developed in the middle and the distal part of the esophagus [17]. In our study, we focused only on the acute esophageal injuries out of the whole upper gastroesophageal tract for the following reasons:

1. The injury to the oropharynx and esophagus was one to two degrees greater than that to the stomach in patients with accidental acid ingestion, possibly because the acids, being bitter, were expelled rapidly [14].

2. Symptoms or even physical examination can be unreliable in determining the severity and extent of injury to the upper gastroesophageal tract due to corrosive intake. Therefore, most of the esophageal injuries go unnoticed.

3. A myth is there that all the corrosive substances hit the stomach directly. Therefore, most of the esophageal injuries go unevaluated unless endoscopy is done.

In our study, we evaluate esophageal injuries by the Zargar classification for acute esophageal injuries due to corrosive intake by considering the endoscopic data of all the patients who underwent endoscopy within the 24 hours of corrosive intake. Timely evaluation of esophageal injury is quite important to prevent complications. Results of our study are different from previous studies in the following aspects:

1. Both upper and lower portions of the esophagus are evaluated separately.

2. Only acute injuries have been considered so that treatment and prognosis can be decided timely and with great precision.

This study has opened doors for other researchers to work further on patterns of esophageal injuries due to corrosive intake and treatment options to improve the prognosis in such patients.

## Conclusions

Most of the patients under study belonged to the female gender and teen and younger age group. The predominant upper esophageal injury as a result of corrosive intake is found to be stage 0 injury (normal mucosa), and the least common is found to be stage 1 injury (superficial mucosal edema and erythema). The predominant lower esophageal injury as a result of corrosive intake is stage 2b injury (deep discrete or circumferential ulcer), whereas the least common is found to be stage 4 injury (perforation). There is statistically no significant relation between age and gender with both upper and lower esophageal injuries.
